# The limitations of mobile phone data for measuring movement patterns of populations at risk of malaria

**DOI:** 10.1186/s12936-025-05416-4

**Published:** 2025-05-31

**Authors:** Greta Tam, Ipsita Sinha, Kulchada Pongsoipetch, Keobouphaphone Chindavongsa, Mayfong Mayxay, Sonexay Phalivong, Benjamin J. Cowling, Olivo Miotto, Supaporn Mahaphontrakoon, Saiamphone Xayvanghang, Richard J. Maude

**Affiliations:** 1 Mahidol Oxford Tropical Medicine Research Unit, Faculty of Tropical Medicine, Mahidol University, Bangkok, 10400 Thailand; 2 WHO Collaborating Centre for Infectious Disease Epidemiology and Control, School of Public Health, Li Ka Shing, Faculty of Medicine, The University of Hong Kong, Hong Kong, China; 3 Centre for Tropical Medicine and Global Health, Nuffield Department of Medicine, University of Oxford, Oxford, OX3 7LG UK; 4 Center of Malariology, Parasitology, and Entomology, Vientiane, Lao People’s Democratic Republic; 5 Lao-Oxford-Mahosot Hospital-Wellcome Trust Research Unit, Microbiology Laboratory, Mahosot Hospital, Vientiane, Lao People’s Democratic Republic; 6 Institute of Research and Education Development, University of Health Sciences, Vientiane, Lao People’s Democratic Republic; 7 Saw Swee Hock School of Public Health, National University of Singapore, Singapore, Singapore; 8 Wellcome Sanger Institute, Hinxton, UK; 9 The Open University, Milton Keynes, MK7 6 AA UK

**Keywords:** Malaria, Mobility, Movement, Risk, Ownership, Usage

## Abstract

**Background:**

As global mobile phone adoption increases, mobile phone data has been increasingly used to measure movement patterns of populations at risk of malaria. However, the representativeness of mobile phone data for populations at risk of malaria has not been assessed. This study aimed to assess this representativeness using prospectively collected data on mobile phone ownership and use from malaria patients in Lao PDR.

**Methods:**

A prospective observational study was conducted from 2017 to 2021. 6320 patients with confirmed malaria in 107 health facilities in the five southernmost provinces of Lao PDR were surveyed regarding their demographics, mobile phone ownership and use. Data on the demographics of mobile phone owners and users in the general population of Lao PDR were obtained from the 2017 Lao Social Indicator Survey II, which was a nationally representative survey sample. Descriptive analysis was performed, and logistic regression with weights on aggregate data was used to compare the demographic distribution of mobile phone ownership and use in malaria patients with that in the general population.

**Results:**

Most patients with malaria (76%) did not own or use a mobile phone. From 2017 to 2021, mobile phone usage in the general population consistently ranged between 53 and 67%, whereas among malaria patients, usage remained significantly lower, fluctuating between 20 and 28%. At the district level, log malaria incidence rate (API) was weakly negatively correlated with the proportion of mobile owners (R^2^ = 0.3, p = 0.005). Mobile phone ownership and usage among malaria patients were significantly lower than in the general population (p-value < 0.001). This trend was consistent across all provinces, suggesting a widespread issue rather than isolated cases. Both male and female malaria patients showed reduced mobile phone access compared to their peers in the general population. Furthermore, this disparity persisted across all age groups, indicating that regardless of age or gender, malaria patients faced barriers to mobile phone ownership and usage. This could have implications for communication and access to health resources, highlighting a critical area for public health interventions.

**Conclusion:**

Mobility data from anonymized and aggregated call data records (CDR) from the general population may not sufficiently represent the population at risk of malaria to accurately model disease transmission. Yet mobile phone data is commonly used to model malaria transmission in endemic countries. Before doing so, it is critical to quantify mobile usage among the population at risk of malaria. Where this is low, either movement estimates derived from mobile phone data need to be adjusted to increase model accuracy, or another method should be used to measure the mobility of populations with malaria.

**Supplementary Information:**

The online version contains supplementary material available at 10.1186/s12936-025-05416-4.

## Background

With recent technological advances, a wide variety of data sources covering the range of spatial and temporal scales have been used to quantify human population movement relevant to the control of malaria. This ranges from daily routine movements in a neighbourhood to international migration [1]. In particular, mobility data from anonymized and aggregated call detail records (CDR) have been increasingly used to measure human population movement (HPM) as global mobile phone adoption increases. In 2020, out of the global population of 7.58 billion people, there were 6.95 billion mobile phone users, with this number projected to rise to 7.49 billion by 2025 [2].

In the last decade, there has been a rapid increase in the number of studies using mobility data to quantify HPM relevant to health. Most of these focused on Low- and Middle-Income Countries (LMIC), where mobile phones are the preferred communication mode due to the lack of landline infrastructure [3]. CDRs can provide data on spatial–temporal HPM through passive data collection, which is much needed in LMIC where this type of data would be difficult to obtain through other methods [4]. In the last 5 years, HPM patterns derived from CDRs have been used to model the transmission of infectious diseases [5], mostly for malaria [4]. During this time, most studies tracking international HPM relevant to the control and elimination of malaria included mobility data in the analyses [1]. Combined with other sources of data, such as malaria prevalence and incidence, source-sink analyses were conducted at different spatiotemporal scales.

Given the widespread uptake of mobile phone use, it is generally assumed that mobility data represents a true random sample of the population being studied [6]. While some studies showed that CDR estimates could accurately replicate population counts and migration patterns when compared to census data [3], low-income countries often have lower and heterogeneous mobile phone ownership rates [7, 8]. Previous studies showed that mobile phone ownership in Rwanda, Kenya, Bangladesh, Pakistan, India, Sri Lanka and Indonesia was not representative of the general population [9–11] and that different sub-populations, such as individuals affected by malaria, may be over or underrepresented due to differential mobile phone ownership [12–15]. Infectious disease models using CDRs are more accurate when modelling highly connected, urban and dense populations [3]. However, the rural poor are often at greater risk of malaria [16]. The digital divide among genders, socioeconomic status, and between urban and rural populations may cause mobile phone-based analyses and models to exacerbate health disparities, particularly in regions where health interventions are most crucial due to existing inequities [17].

To assess the representativeness of CDRs, it is necessary to consider the fraction of the population represented by mobile phone owners and users, as well as the demographic and geographical representativeness. Characteristics of mobile phone owners and users can be compared to a public gold standard, such as a census [18]. Assessing whether biases in these areas exist will enable an understanding of how limitations affect modelling results. Estimates could then be adjusted to increase model accuracy [3].

There is a lack of data assessing the representativeness of CDRs for populations at risk of malaria. It has also been suggested that mobile phone sharing has led to an exaggeration of the digital divide between developed and developing countries, with surveys in multiple African countries showing that phones were commonly shared within the household and community, among five people on average in Ethiopia, for example [6]. The study aimed to evaluate the representativeness of mobile phone ownership by analysing prospectively collected survey responses from malaria patients in Lao PDR (a LMIC). It compared the characteristics of mobile phone owners and users to publicly available survey data from the general population, while also describing the patterns of mobile phone use and ownership among these malaria patients.

## Methods

### Field study

A prospective observational study was conducted, as part of GenRe-Mekong, a genetic surveillance project. A total of 6320 patients who self-presented, tested positive for malaria by microscopy and/or rapid diagnostic test and provided informed consent were enrolled at 107 health facilities across 5 contiguous provinces in Southern Lao People’s Democratic Republic (Lao PDR): Attapeu, Champasak, Saravane, Savannakhet and Sekong. Face-to-face interviews were conducted using the established protocols of the GenRe-Mekong genetic surveillance project [19–21]. Data was collected on paper form by the research team. Ethical approvals were obtained from the National Ethics Committee for Health Research of the Health Ministry of the Lao People's Democratic Republic and the Oxford Tropical Research Ethics Committee.

### Comparison data and sample

Data on the demographics of mobile phone owners and users in the general population of Lao PDR were obtained from the 2017 Lao Social Indicator Survey II [22], which was a nationally representative survey sample. The survey sample included 26,103 women (age 15–49) and 12,694 men (age 15–49) from 23,299 households. Response rates were 96.9% for women and 94.7% for men. A two-stage sample design was used. In stage 1, the urban and rural areas within each province were identified as the main sampling strata. Within each stratum, census enumeration areas were systematically selected with probability proportional to size. In stage 2, a systemic sample of 20 households was drawn from each area.

### Other sources of data

Data on the temporal trend of mobile phone use in the general population was sourced from the World Bank [23]. Data on the proportion of mobile users having internet on their phone in the general population was sourced from DataReportal [24]. Data on the market share of mobile phone providers in the general population was sourced from the Asia–Pacific Telecommunication Policy and Regulatory Forum [25]. Ranges of malaria API of districts in Lao PDR were sourced from the World Health Organization (WHO) [26]. The upper bounds of the ranges were used.

### Analyses

Demographics of respondents, mobile service providers used, whether they had internet on their phone, number of people they shared a phone with, days per week they carried a mobile phone, number of SIM cards they owned and how often they used their phone were reported using descriptive statistics. To compare the demographic distribution of mobile phone ownership and use in malaria patients with those in the general population, logistic regression with weights on aggregate data was used. The primary outcome variable was mobile phone ownership and use. Independent variables were age, gender and province. The level of significance used was p < 0.05. All statistics were analysed using R 4.0.3.

## Results

The dataset included 6320 individuals with malaria from May 2017 to December 2021 (Supplementary Table 1), with 1720 females and 4595 males. The median (range) age was 19 (0–90) years. Individuals from Attapeu numbered 1979, Champasak had 655, Saravane had 968, Savannakhet accounted for 1611, and Sekong contributed 1010. Among the cases, 3688 were identified as *Plasmodium vivax*, 2559 as *Plasmodium falciparum*, 67 had mixed infections of *P. falciparum* and *P. vivax*, and there was 1 case each with *Plasmodium knowlesi* and *Plasmodium ovale*.

### Mobile phone ownership and use

A significant portion of individuals, specifically 4817 (76%), did not own a mobile phone, and 4792 (76%) reported that they did not use one. These proportions were similar across subgroups, so only usage is presented through the remainder of the Results.

**Fig. 1:**
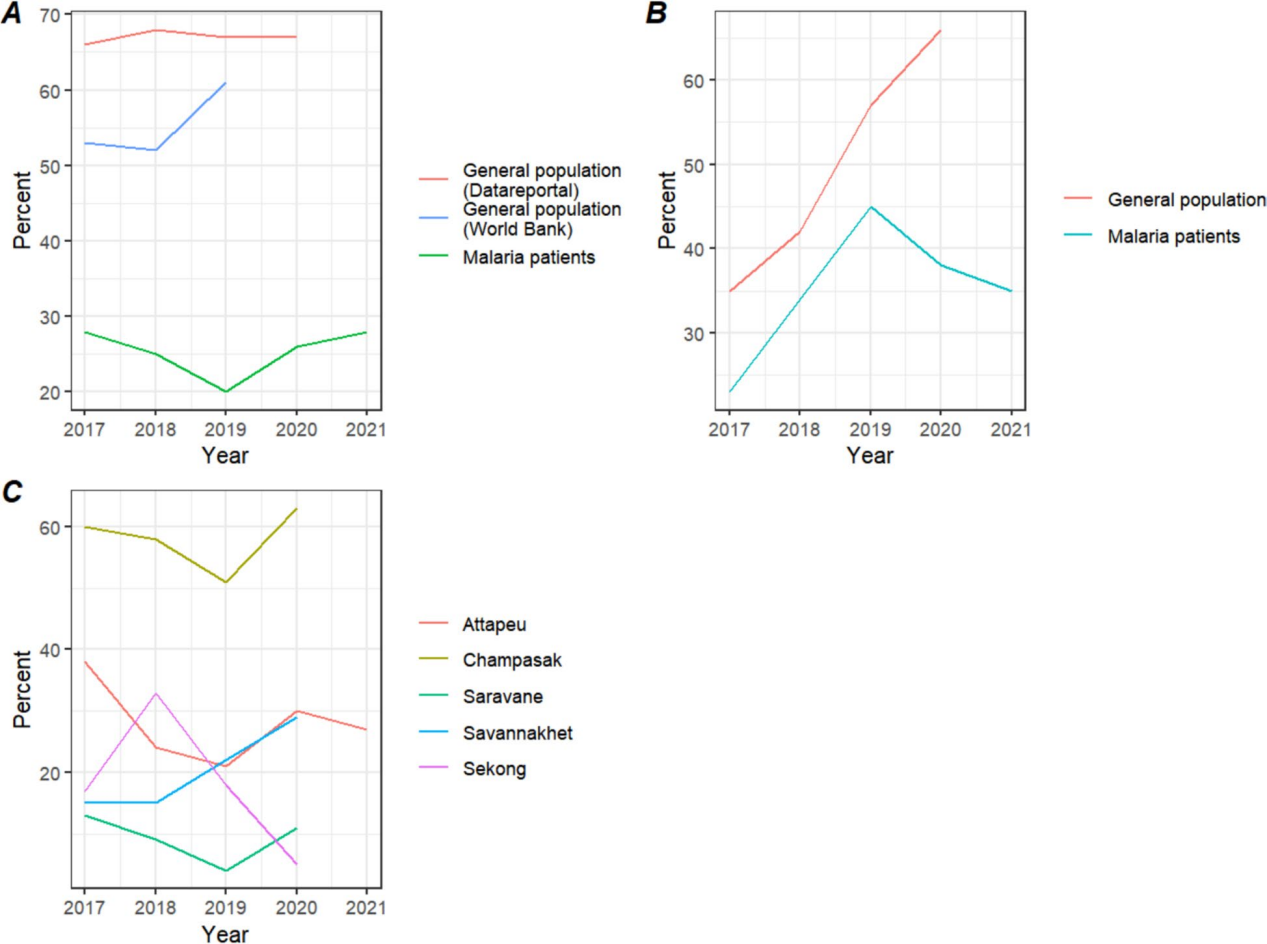
Trends of mobile phone usage by year from 2017 to 2021. **A** Mobile phone use from 2017 to 2021 amongst the general population and malaria patients. **B** Mobile users having internet from 2017 to 2021 in the general population and malaria patients. **C** Mobile phone use across provinces among malaria patients

While mobile phone use in the general population remained static at median 67% (range 66–67%) (according to DataReportal) and median 55% (53–56%) (according to the World Bank), mobile phone use in malaria patients was much lower and generally remained the same over the years, with median 25% (range 20% to 28%) (Fig. 1A). The lower mobile phone ownership and usage among malaria patients was consistently observed across both genders (p < 0.001), in each province, within each gender across provinces, across all age groups, and within each age group when further divided by gender (p < 0.001) (Supplementary Tables 1–6).

The proportion of mobile users with internet on their phone was lower for malaria patients at a median 38% (range 23% to 56%) than in the general population at 57% (35% to 76%). Over time, it increased for both, from 35% in 2017 to 66% in 2021 for the general population and from 23 to 56% for malaria patients (Fig. 1B).

Mobile phone use was much higher overall in Champasak province at a median 58% (range 60–63%) than in the others at a median 17.5 (range 4% to 38%). Mobile phone use decreased from 2017 to 2019 and increased again from 2019 to 2020 in Champasak, Attapeu and Saravane provinces. In Attapeu and Saravane, it plateaued from 2020 to 2021. In Savannakhet, it was static from 2017 to 2018, increased from 2018–2020, and then decreased from 2020 to 2021. In Sekong, it increased from 2017 to 2018, decreased from 2018 to 2020 and plateaued from 2020 to 2021 (Fig. 1C).

**Fig. 2:**
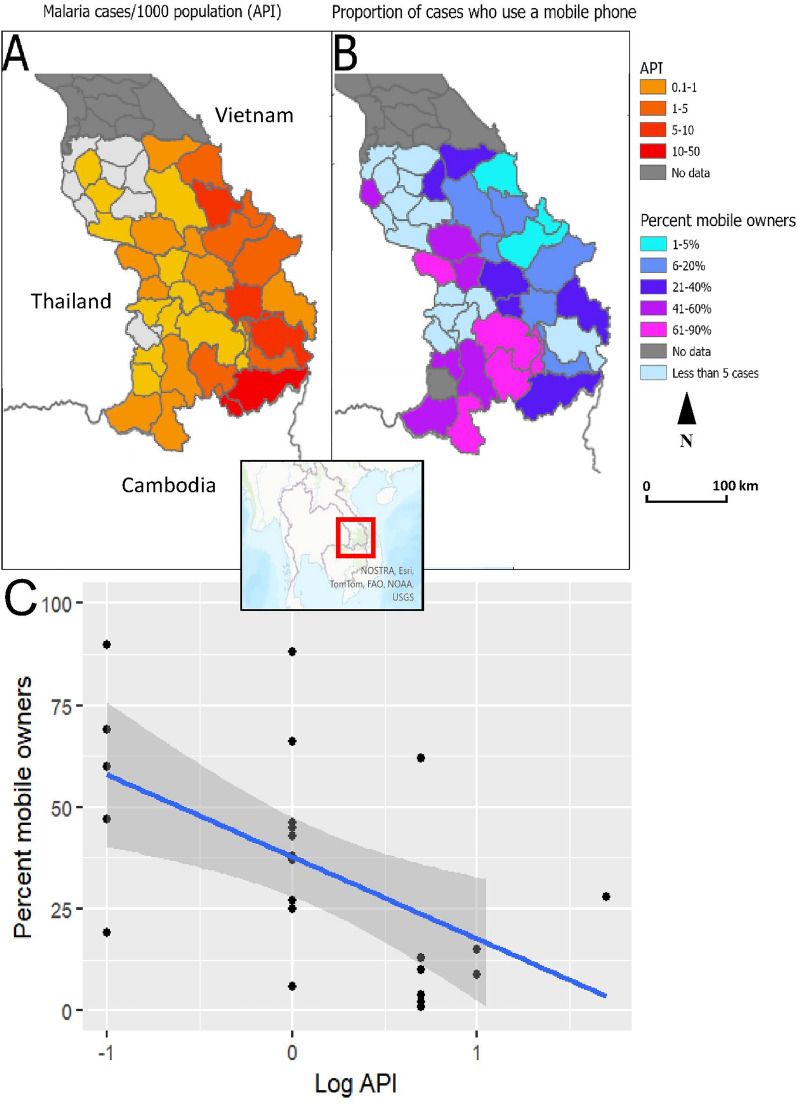
Malaria incidence and mobile phone use. **A** Maps of malaria incidence and **B** proportion of malaria patients who were mobile phone owners in 2017–2021 by district in Lao PDR and **C** scatterplot of logarithmically transformed malaria incidence and percentage malaria patients who own a mobile phone

At the district level, log malaria incidence rate (API) was weakly negatively correlated with the proportion of mobile owners (R^2^ = 0.3, p-value = 0.005) (Fig. 2).

**Fig. 3:**
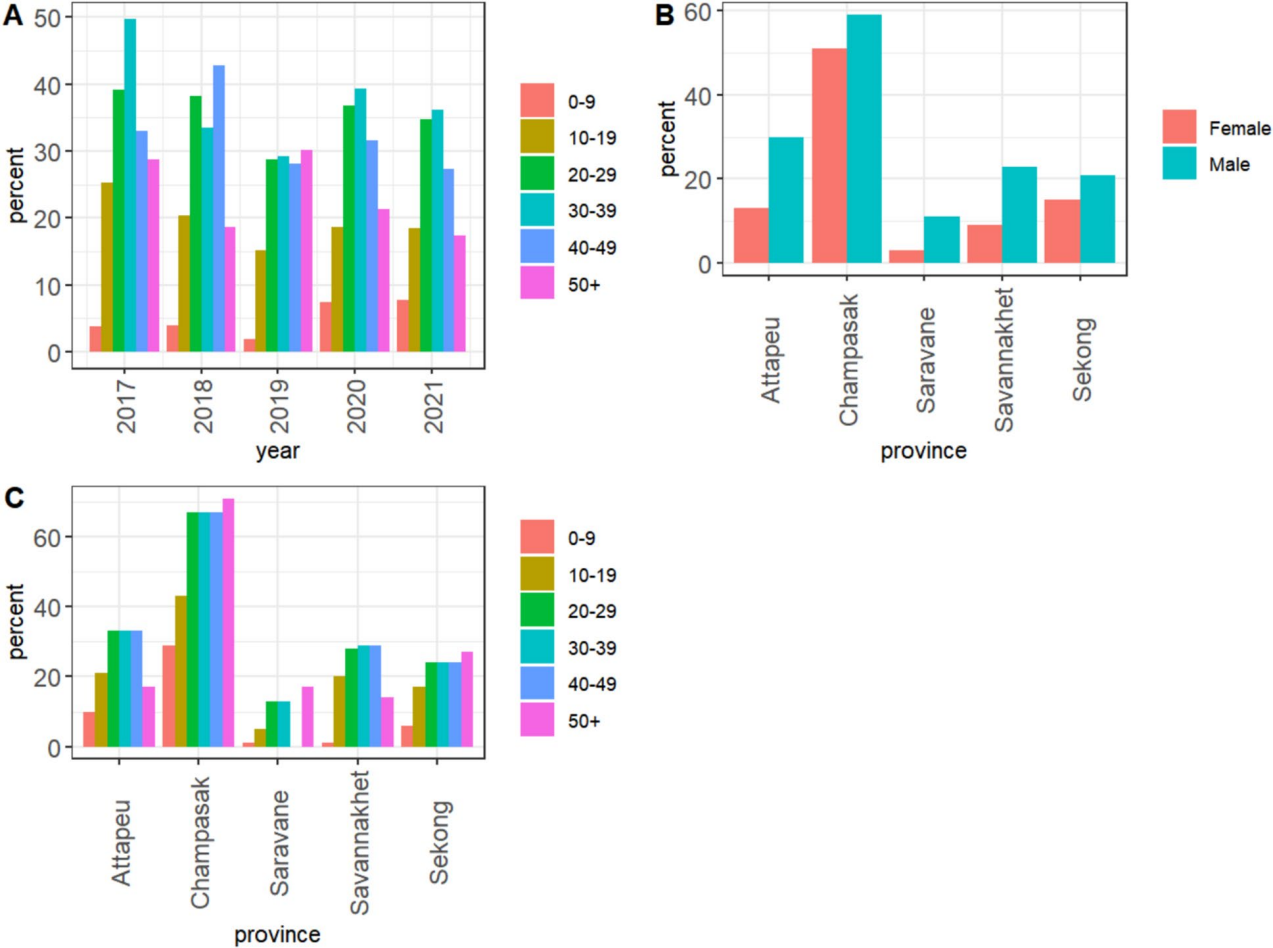
Demographics and mobile phone use by malaria patients. **A** Mobile phone use by age group and year. **B** Mobile phone use by gender and province. **C** Mobile phone use by age group and province

Mobile phone use was highest in the 30–39-year age group (37%) and higher in males (29%) than in females (12%, p < 0.001). Over the years, among malaria patients aged 10–49 years, mobile phone use decreased from 2017 to 2019, increased from 2019 to 2020, and then decreased slightly in 2021. Usage remained constant among the 0–19 years age group in 2021 and generally declined in other age groups (Fig. 3A).

Among malaria patients, the proportion of mobile users by gender differed by province, Saravane had the highest ratio of males to females (3.7) and Champasak the lowest (1.2) (Fig. 3B). The age distribution of mobile users differed by province. Champasak, Saravane and Sekong had proportionately more users over 50 years of age than Attapeu and Savannakhet (Fig. 3C).

**Fig. 4:**
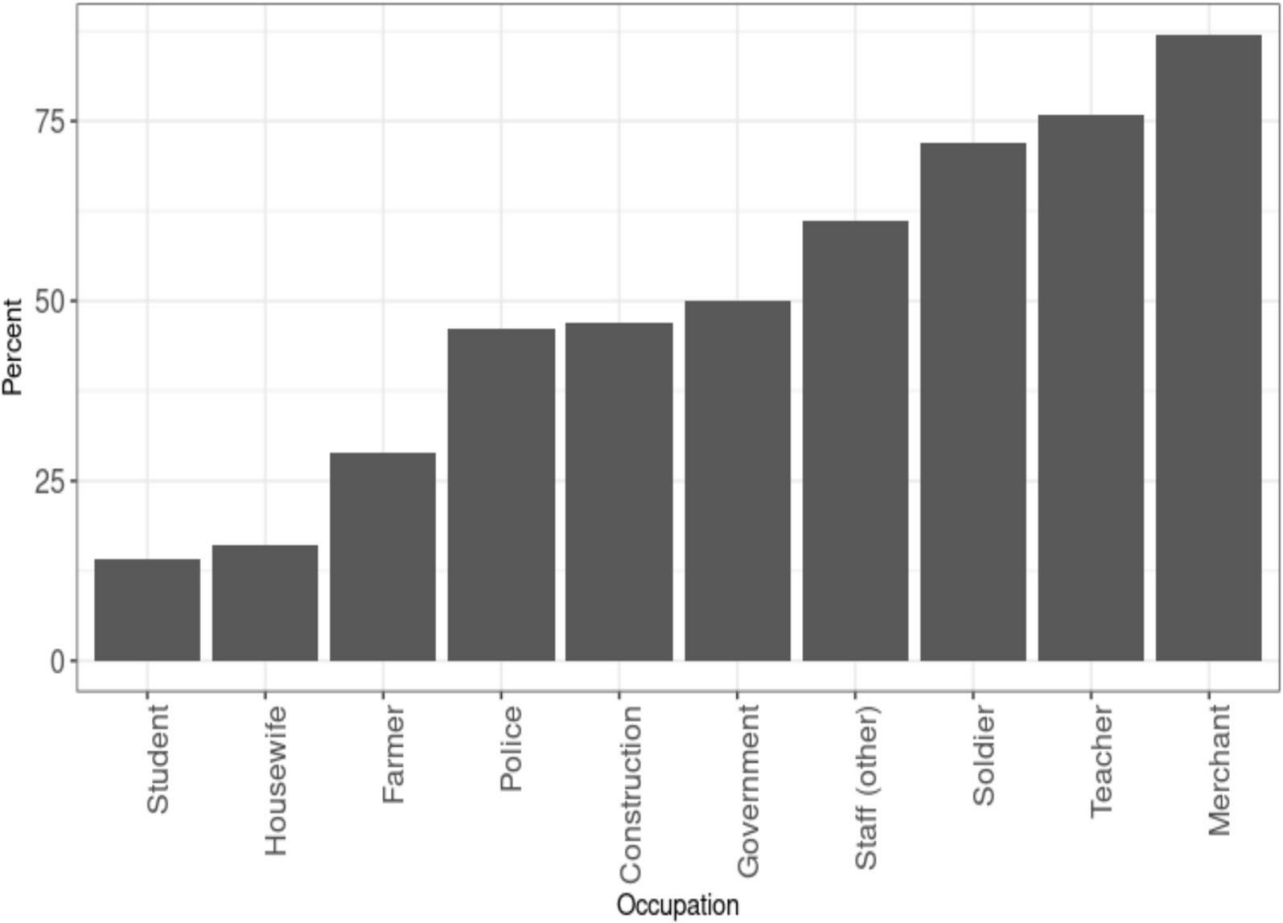
Percent of malaria patients with the top 10 occupations using a mobile

Amongst the top 10 most frequently reported occupations, merchants, teachers and soldiers, had the highest proportions using mobile phones, whilst students, housewives, and farmers had the lowest (Fig. 4). Mobile use was highest among soldiers and teachers in Attapeu and Champasak, soldiers in Saravane and Savannakhet and construction workers in Sekong (Supplementary Fig. 1).

**Fig. 5:**
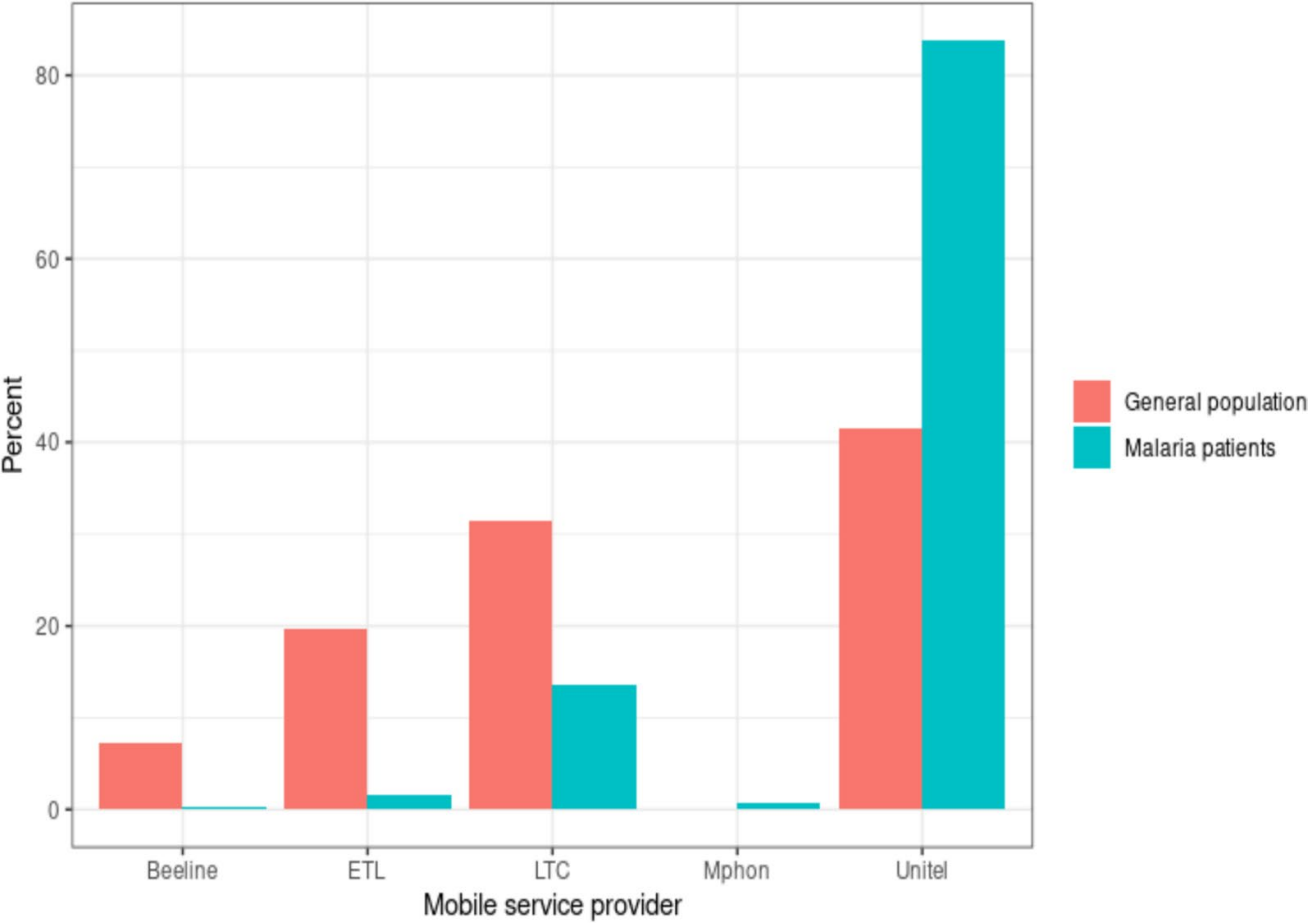
Market share of mobile phone service providers nationally, amongst mobile phone owners in the general population and malaria patients

Market share was more evenly distributed among mobile phone providers in the general population than amongst malaria patients. Although Unitel was the most popular in both groups, a much higher proportion (84% vs 42%) of malaria patients chose Unitel as their mobile provider (Fig. 5).

**Fig. 6:**
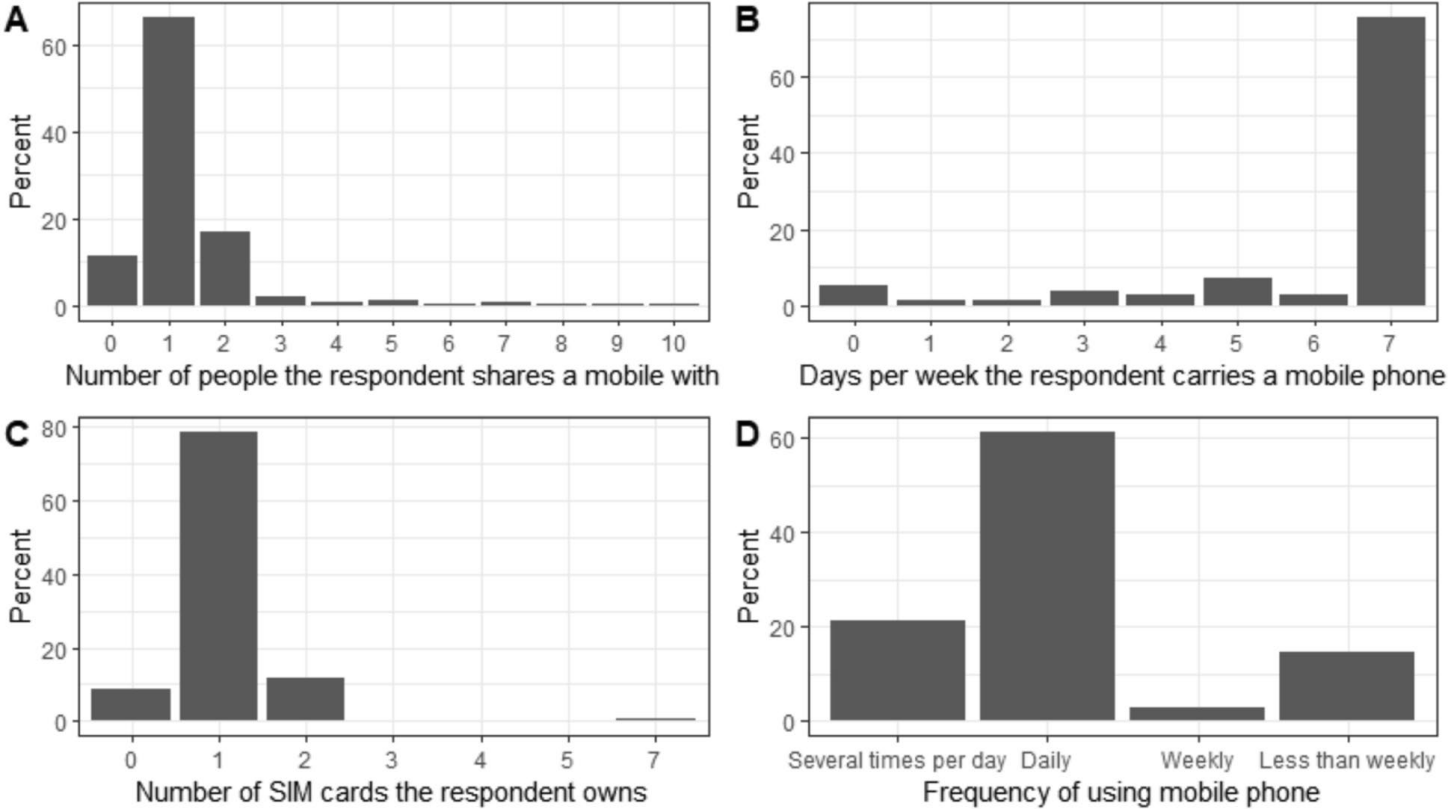
Mobile phone usage behaviours among malaria patients. **A** Percentage of mobile phone users who shared a mobile by the number of people they share it with. **B** Number of days per week mobile phone users reported carrying a mobile phone. **C** Number of SIM cards that mobile phone users owned. **D** Frequency of mobile phone use amongst malaria patients

Among mobile phone users, 66% shared their device with one person, 17% with two people, 6% with more than two, and 11% did not share at all (Fig. 6A). 61% of these users carried their mobile phone daily (Fig. 6B). Additionally, 79% of mobile phone users owned a single SIM card. (Fig. 6C). Overall, 61% of mobile phone users used their phone daily (Fig. 6D).

Mobile phone sharing differed by province. Savannakhet had the lowest proportions of users not sharing and sharing with two people (5% and 7% respectively) and the highest proportion sharing with one person (84%), while Saravane had the highest proportion of users not sharing and sharing with two people (16% and 29%, Supplementary Fig. 2).

In Attapeu, 29% of people utilized their mobile phones several times a day, whereas only 13% did so in Savannakhet. Daily mobile phone usage was reported by 80% of individuals in Savannakhet, compared to 46% in Sekong. In Saravane, 61% of people used their phones weekly, while Savannakhet had no users in this category. Additionally, 38% of individuals in Sekong used their mobile phones less than once a week, in contrast to just 7% in Savannakhet. (Supplementary Fig. 3).

## Discussion

This study is one of the first to quantify differences in mobile phone ownership and usage among malaria patients. It uses data specifically collected for this purpose and compares it with mobile phone ownership in the general population based on existing survey data. To date, studies analysing human population movement data for malaria control and elimination have used CDRs from the general population obtained from mobile phone providers, in combination with other sources of data specific to a population at-risk of malaria [1]. The present study provides a detailed picture of demographic factors influencing mobile phone ownership and use in a population at-risk of malaria, as well as showing patterns of use and ownership.

After comparing the demographics of mobile phone users and owners in malaria patients with the general population, it is clear that there is a digital divide between the general population in Lao PDR (a LMIC) and populations at risk of malaria that persists across demographic groups and geographical regions.

Basic mobile ownership in the Lao PDR general population already lags behind neighbouring countries, including other ASEAN countries, Vietnam and Cambodia [27]. Phone use may have remained persistently low over the years due to numerous obstacles: Mobile phone services are costly and relatively unaffordable in Lao PDR [27]. In addition, there may be a lack of access to electricity and low literacy [28]. The present study’s findings of a digital divide between malaria patients and the general population within the same country as well as a lower proportion of mobile owners in districts where malaria incidence was highest would fit with malaria being a disease of poverty [28] with predominant transmission in rural areas [29] and this may be contributing to the further internal widening of the digital divide. Worryingly, the present study showed that this gap occurs across all genders, age groups and provinces in Lao PDR. Thus, there is a limited representativeness when using CDRs from a general population to model transmission in populations at-risk of malaria. In addition, using mobile health (mHealth) technologies to improve the health of populations at-risk of malaria may result in further disparities for a large proportion of this group that already faces existing barriers to access. This is a concern already expressed by the WHO, which acknowledged potential access, acceptability and affordability difficulties in using mHealth in target communities in LMIC [30]. A study in rural Saravane province in Lao PDR concluded that although mHealth resulted in faster access to public healthcare, it could crowd out local social support networks in rural settings, thereby increasing inequality [31].

Mobile internet use in Lao PDR lags behind other ASEAN countries, Vietnam, Cambodia and Myanmar, likely due to the high cost and low coverage [27]. In 2017, 91% of the general population had access to a 2G network, while only 65% and 13% had access to 3G and 4G networks, respectively [27]. The present study showed that in Lao PDR, there was a smaller digital divide between malaria patients and the general population for mobile internet use, compared to basic mobile ownership. The digital divide refers to the disparity in mobile ownership and internet access between malaria patients and the general population in Lao PDR. This may represent a subset of people in both populations who can afford mobile internet use, and overcome technological, literacy and connectivity barriers [32]. Access to 2G, 3G, or 4G networks is crucial for malaria-positive patients as it enables them to access vital health information, telemedicine services, and educational resources. Enhanced data usage can facilitate timely communication with healthcare providers, improve adherence to treatment, and promote awareness about malaria prevention and management strategies. Mobile health (mHealth) initiatives have shown that improved connectivity significantly enhances patient outcomes by enabling real-time data sharing and faster intervention for malaria cases [33, 34].

This study showed that an overwhelming majority of malaria patients used Unitel as their mobile phone provider, while mobile phone provider choice was more evenly spread out across the general population. As Unitel’s mobile network covers 95% of the population [35], this may represent a monopoly of the market and less competitive prices in southern Lao PDR compared to the rest of the country. Unitel's monopoly may lead to higher service costs, potentially limiting communication for malaria positive patients already burdened by medical expenses. A possible upside to this monopoly is that analysis of CDR for mobility of malaria patients in Lao PDR may only need to access data from this one provider, enabling targeted interventions and improved monitoring of patient mobility. Partnerships between health programmes and Unitel could enhance communication through tailored health alerts and educational resources, while advocacy for competitive pricing is essential to ensure equitable access for vulnerable populations. Ultimately, leveraging Unitel’s network presents both challenges and opportunities to improve health outcomes for malaria patients.

While mobile phone use in most provinces in Lao PDR followed the temporal trend of use in malaria patients as a whole, Savannakhet’s trend was more similar to that of the general population, and mobile phone use in Champasak was higher, similar to the general population. Sekong had a unique trend, with steadily decreasing mobile phone use since 2018 and the least frequent use of mobile phones compared to other provinces. Across all age groups and genders, Champasak had the highest mobile phone use, while Saravane had the lowest. Provincial differences in poverty and population size may be contributing factors. Both Savannakhet and Champasak have a large population size, although the poverty rate is high in Savannakhet and low in Champasak. In Savannakhet, despite a large population and high poverty rates, mobile usage mirrors that of the general population, suggesting that while access may be widespread, economic barriers could limit effective use, particularly for health-related services. Conversely, Champasak, with lower poverty rates and higher mobile usage, may provide a more conducive environment for accessing health information and services, potentially leading to better health outcomes for malaria patients.

Sekong has a small population size and the highest poverty rate in Lao PDR, while a large proportion of the poor reside in Saravane [32]. A large population may lead to companies providing better mobile phone coverage. Sekong presents a contrasting scenario to Savannakhet and Champasak, with a steady decline in mobile usage since 2018, likely exacerbated by its small population size and the highest poverty rate in the country. This reduction in connectivity may hinder malaria-positive patients in Sekong from accessing crucial health information and support, impacting their treatment adherence and overall health management. Similarly, Saravane, with the lowest mobile phone use, indicates that a large portion of its impoverished population lacks access to mobile services, further complicating the fight against malaria. These provincial differences highlight the need for tailored public health strategies. In regions with lower mobile usage, such as Sekong and Saravane, programmes should focus on improving mobile connectivity and promoting affordable plans, enabling better access to health resources. In contrast, areas like Champasak could leverage their higher mobile usage to implement targeted health initiatives, utilizing SMS alerts and mobile health applications to enhance malaria management. Overall, understanding these trends and their associations with socio-economic factors is crucial for developing effective malaria control programmes that address regional disparities and improve health outcomes for affected populations.

The finding that amongst malaria patients, wage and salaried workers (employees) [27] tended to own mobile phones is consistent with a Pew Research Center report, which listed cost and lack of technological and general literacy as barriers to owning mobile phones for people in emerging economies [28]. However, occupation may not be the only factor contributing to mobile phone use, as mobile phone use amongst the top five occupations still varied by province.

This study showed that in Lao PDR, although only a small proportion of mobile phone users and owners did not share their phones, most only shared them with one person. Thus, it is unlikely that the digital divide has been vastly overestimated. However, this sharing behaviour has implications for how CDRs are interpreted in that one record may represent travel of more than one person. In addition, most malaria patients who owned or used a mobile phone carried and used theirs daily. An overwhelming majority owned only one SIM card, in contrast to other developing countries [28]. This has the advantage of CDR from a single SIM being more representative of the overall travel patterns of users as compared to countries where SIMs might be swapped due to, for example, variation in signal strength from different service providers. This study did not examine the geographical coverage of mobile phone services, but this is often much lower in rural areas where malaria is typically found. A previous study in Bangladesh combined travel survey and mobile phone CDR to give better geographic coverage to analyse the impact of mobility on malaria [36]. The mobile phone usage patterns among malaria-positive patients in Lao PDR present both challenges and opportunities for health interventions. To improve health outcomes, public health programmes should leverage CDR data for targeted interventions, integrate mobility data with health surveys, and address connectivity gaps in underserved regions. By harnessing mobile technology effectively, health initiatives can enhance communication, track patient movements, and ultimately contribute to more effective malaria control strategies.

In summary, this study demonstrates the need to quantify mobile phone usage in a population to assess the representativeness of mobile phone data in that population. The low usage seen among malaria patients in Lao PDR is likely to be mirrored in malaria-affected populations in other countries around the world. Mobile phone data should not be used as a proxy for mobility of populations affected by communicable diseases without also quantifying usage of mobile phones by those affected populations. Then, where usage is low, it can be decided whether to adjust for this in the analysis or to use another more representative measure of mobility.

## Conclusions

In conclusion, mobile phone usage among malaria-affected individuals in Lao PDR is significantly lower than in the general population, which raises concerns about the representativeness of CDRs for modelling malaria transmission. This limited usage introduces biases that could adversely affect the accuracy of mathematical modelling results in other studies. To enhance the reliability of estimates derived from CDR data, adjustments must be made to account for these biases, or alternative measures of population mobility should be employed.

Furthermore, the integration of mHealth technologies aimed at improving health outcomes for at-risk populations could inadvertently exacerbate healthcare access issues for those already facing barriers. Therefore, public health programmes must focus on enhancing mobile connectivity, particularly in rural areas, and ensure inclusive strategies that address the specific needs of malaria-positive patients. By doing so, health initiatives can effectively leverage mobile technology to support better health outcomes and more accurate malaria control efforts in Lao PDR.

## Supplementary Information

Additional file1

## Data Availability

The dataset generated and analysed during the current study is not publicly available as it belongs to the Center of Malariology, Parasitology, and Entomology (CMPE) and Mahidol Oxford Tropical Medicine Research Unit. Raw identifiable spatial data cannot be shared but aggregated format may be shared upon reasonable request after discussion and agreement from CMPE and contingent upon approval by the MORU Data Access Committee (datasharing@tropmedres.ac). Data on the demographics of mobile phone owners and users in the general population of Lao PDR are available from the 2017 Lao Social Indicator Survey II [33]. Data on the temporal trend of mobile phone use in the general population are available from the World Bank [19]. Data on the proportion of mobile users having internet on their phone in the general population are available from DataReportal [20]. Data on the market share of mobile phone providers in the general population are available from the Asia–Pacific telecommunity policy and regulatory forum [21]. Data on malaria API of districts in Lao PDR area available from the WHO [22]. The Lao PDR administrative boundaries dataset used in the current study is publicly available at Humanitarian Data Exchange (https://data.humdata.org/dataset/cod-ab-lao?).
