# Supplementary material for: The limitations of mobile phone data for measuring movement patterns of populations at risk of malaria
Source: Malar J. 2025 May 31;24:174. doi: 10.1186/s12936-025-05416-4 (PMC12126860; doi:10.1186/s12936-025-05416-4)
Supplement: Supplementary file 1 — Additional file1 [file 12936_2025_5416_MOESM1_ESM.docx]

**Supplementary figures and tables**


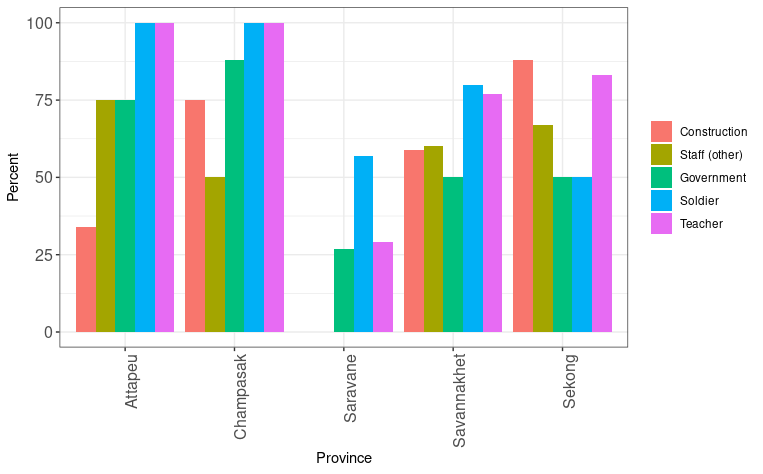


Supplementary figure 1. Proportion of people with the top five occupations using a mobile phone, among malaria patients by province


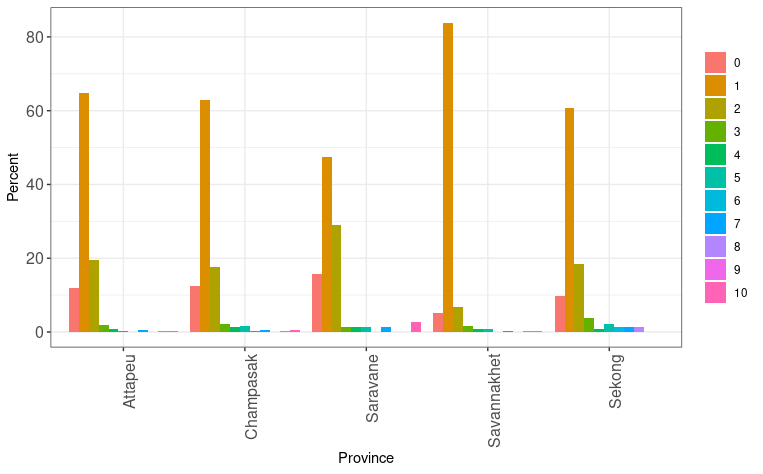


Supplementary figure 2. The percentage of mobile phone users or owners amongst malaria patients who share a mobile and the number of people they share a mobile phone with, by province.


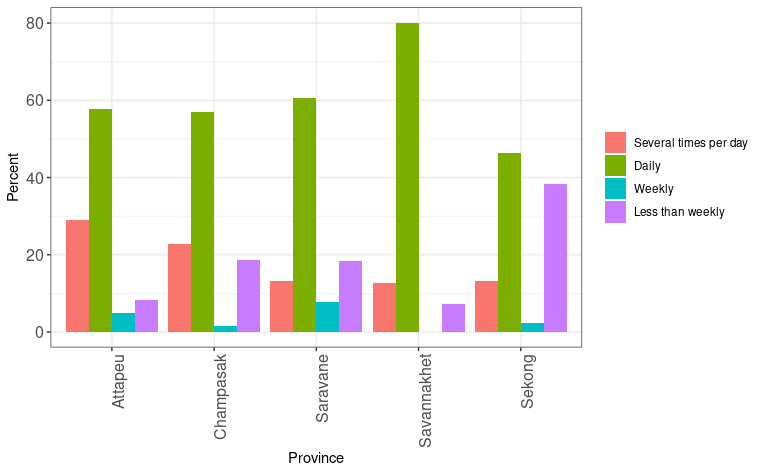


Supplementary figure 3. Frequency of mobile phone use in malaria patients, by province

| Year | Number recruited |
| --- | --- |
| 2017 | 1201 |
| 2018 | 1782 |
| 2019 | 1462 |
| 2020 | 1049 |
| 2021 | 826 |
| Total | 6320 |

Supplementary table 1. Malaria patients recruited by year

|  | Mobile phone ownership by malaria patients | Mobile phone ownership in 2017 census |
| --- | --- | --- |

| Gender | Sample size | % | Sample size | % | P-value |
| --- | --- | --- | --- | --- | --- |
| Female | 1412 | 12 | 25305 | 73.1 | <0.001 |
| Male | 3992 | 28 | 12017 | 84.3 | <0.001 |

|  | Mobile phone use in malaria patients | Mobile phone use in 2017 census |
| --- | --- | --- |

| Gender | Sample size | % | Sample size | % |  |
| --- | --- | --- | --- | --- | --- |
| Female | 1412 | 12 | 25305 | 79.6 | <0.001 |
| Male | 3993 | 29 | 12017 | 87 | <0.001 |

Supplementary table 2. Comparison of gender in mobile phone ownership and use in malaria patients with the general population of Lao PDR from the 2017 Lao Social Indicator Survey II.

|  | Mobile phone ownership in female malaria patients | | Mobile phone ownership amongst females in 2017 census | | |
| --- | --- | --- | --- | --- | --- |
| Province | Sample size | % | Sample size | % | P-value |
| Attapeu | 318 | 12 | 541 | 67.5 | <0.001 |
| Champasak | 100 | 50 | 2396 | 78.5 | <0.001 |
| Saravane | 306 | 3 | 1510 | 52.5 | <0.001 |
| Savannakhet | 443 | 8 | 3351 | 61.6 | <0.001 |
| Sekong | 240 | 15 | 431 | 52.1 | <0.001 |
|  | Mobile phone use by female malaria patients | | Mobile phone use amongst females in 2017 census | | |
| Province | Sample size | % | Sample size | % | P-value |
| Attapeu | 318 | 13 | 541 | 76.6 | <0.001 |
| Champasak | 100 | 51 | 2396 | 86.4 | <0.001 |
| Saravane | 306 | 3 | 1510 | 54.6 | <0.001 |
| Savannakhet | 443 | 9 | 3351 | 67.4 | <0.001 |
| Sekong | 240 | 15 | 431 | 55.3 | <0.001 |

Supplementary table 3. Comparison of mobile phone ownership and use by province in female malaria patients with the female population of Lao PDR from the 2017 Lao Social Indicator Survey II.

|  | Mobile phone ownership in female malaria patients | | Mobile phone ownership amongst females in 2017 census | | |
| --- | --- | --- | --- | --- | --- |
| Age (years) | Sample size | % | Sample size | % | P-value |
| 0-15 | 713 | 5 | - | - | - |
| 15-19 | 176 | 16 | 4565 | 74.8 | <0.001 |
| 20-24 | 124 | 14 | 4024 | 77.2 | <0.001 |
| 25-29 | 103 | 21 | 4045 | 74.4 | <0.001 |
| 30-34 | 88 | 22 | 3824 | 73.8 | <0.001 |
| 35-39 | 68 | 24 | 3418 | 69.2 | <0.001 |
| 40-44 | 39 | 15 | 3076 | 68.7 | <0.001 |
| 45-49 | 58 | 22 | 2353 | 71.2 | <0.001 |
| 50+ | 80 | 18 | - | - | - |

Supplementary table 4. Comparison of age of mobile phone ownership and use in female malaria patients with the female population of Lao PDR from the 2017 Lao Social Indicator Survey II.

|  | Mobile phone ownership in male malaria patients | | Mobile phone ownership amongst males in 2017 census | | |
| --- | --- | --- | --- | --- | --- |
| Province | Sample size | % | Sample size | % | P-value |
| Attapeu | 1314 | 30 | 243 | 80.1 | <0.001 |
| Champasak | 561 | 59 | 1119 | 84.8 | <0.001 |
| Saravane | 622 | 11 | 709 | 80.1 | <0.001 |
| Savannakhet | 974 | 23 | 1619 | 72.4 | <0.001 |
| Sekong | 476 | 20 | 209 | 70.4 | <0.001 |
|  | Mobile phone use in male malaria patients | | Mobile phone use amongst males in 2017 census | | |
| Province | Sample size | % | Sample size | % | P-value |
| Attapeu | 1314 | 30 | 243 | 86.3 | <0.001 |
| Champasak | 561 | 59 | 1119 | 88.5 | <0.001 |
| Saravane | 622 | 11 | 709 | 82.7 | <0.001 |
| Savannakhet | 975 | 23 | 1619 | 72.1 | <0.001 |
| Sekong | 476 | 21 | 209 | 70.2 | <0.001 |

Supplementary table 5. Comparison of locations of mobile phone ownership and use in male malaria patients with the male population of Lao PDR from the 2017 Lao Social Indicator Survey II.

|  | Mobile phone ownership in male malaria patients | | Mobile phone ownership amongst males in 2017 census | | |
| --- | --- | --- | --- | --- | --- |
| Age (years) | Sample size | % | Sample size | % | P-value |
| 0-15 | 80 | 17 | - | - | - |
| 15-19 | 691 | 31 | 2404 | 76.1 | <0.001 |
| 20-24 | 513 | 38 | 1774 | 84.5 | <0.001 |
| 25-29 | 482 | 39 | 1810 | 85.2 | <0.001 |
| 30-34 | 356 | 42 | 1772 | 87.9 | <0.001 |
| 35-39 | 300 | 38 | 1612 | 86.6 | <0.001 |
| 40-44 | 202 | 44 | 1412 | 87.4 | <0.001 |
| 45-49 | 107 | 35 | 1232 | 87.4 | <0.001 |
| 50+ | 188 | 25 | - | - | - |
|  | Mobile phone use in male malaria patients | | Mobile phone use amongst males in 2017 census | | |
| Age (years) | Sample size | % | Sample size | % | P-value |
| 0-15 | 1293 | 10 | - | - | - |
| 15-19 | 692 | 31 | 2404 | 80.3 | <0.001 |
| 20-24 | 513 | 38 | 1774 | 86.9 | <0.001 |
| 25-29 | 482 | 40 | 1810 | 87.8 | <0.001 |
| 30-34 | 356 | 43 | 1772 | 89.1 | <0.001 |
| 35-39 | 300 | 38 | 1612 | 89.5 | <0.001 |
| 40-44 | 202 | 44 | 1412 | 90.1 | <0.001 |
| 45-49 | 107 | 33 | 1232 | 89.8 | <0.001 |
| 50+ | 188 | 25 | - | - | - |

Supplementary table 6. Comparison of age of mobile phone ownership and use in male malaria patients with the male population of Lao PDR from the 2017 census
